# Isolated Central Sulcus Hemorrhage: A Rare Presentation Most Frequently Associated with Cerebral Amyloid Angiopathy

**DOI:** 10.1155/2012/574849

**Published:** 2012-12-11

**Authors:** Murthy R. Chamarthy, Yogesh Kumar, Michael D. Meszaros, Ankit Shah, Mark A. Rosovsky

**Affiliations:** Department of Radiology, Bridgeport Hospital, Yale New Haven Health System, 267 Grant Street, Bridgeport, CT 06610, USA

## Abstract

Central sulcus hemorrhage is a rare imaging finding that can be related to cerebral amyloidosis in a normotensive non-traumatic elderly patient and present as an isolated finding or in association with other areas of involvement. We report a case presenting with an isolated central sulcus hemorrhage on computed tomography. Further imaging work-up excluded other potential causes of peripheral hemorrhages and established a putative diagnosis of cerebral amyloidosis.

## 1. Introduction

Intracranial hemorrhage could be located within the deep white matter, cortical/subcortical, subarachnoid, subdural, epidural, or intraventricular locations. The most common etiologies for intracranial bleeding include trauma and hypertension. Cerebral amyloid angiopathy (CAA) is a common cause of non-traumatic peripheral intracranial hemorrhage in a normotensive patient, and may present as an isolated cortical or subcortical hemorrhage. Imaging evaluation supplements the nonspecific clinical features and helps to characterize the etiology of the hemorrhage and associated complications. Prompt diagnosis of acute as well as chronic presentations of cerebral amyloidosis is important and would result in appropriate management and care.

## 2. Case Report

An 84-year-old woman with history of breast cancer presents to the emergency department with intermittent numbness and paresthesias of the right upper and lower extremities for two days. There is no associated headache, vertigo, or vision problem during the course of illness. There is no history of trauma. There were no constitutional symptoms or chest pain and palpitations. Patient denied any history of unusual bruising, bleeding, or coagulation problems. There is no relevant family history. Vitals signs were stable. Neurological examination was unremarkable without any sensory or motor deficits. The laboratory values were normal.

**Figure 1:**
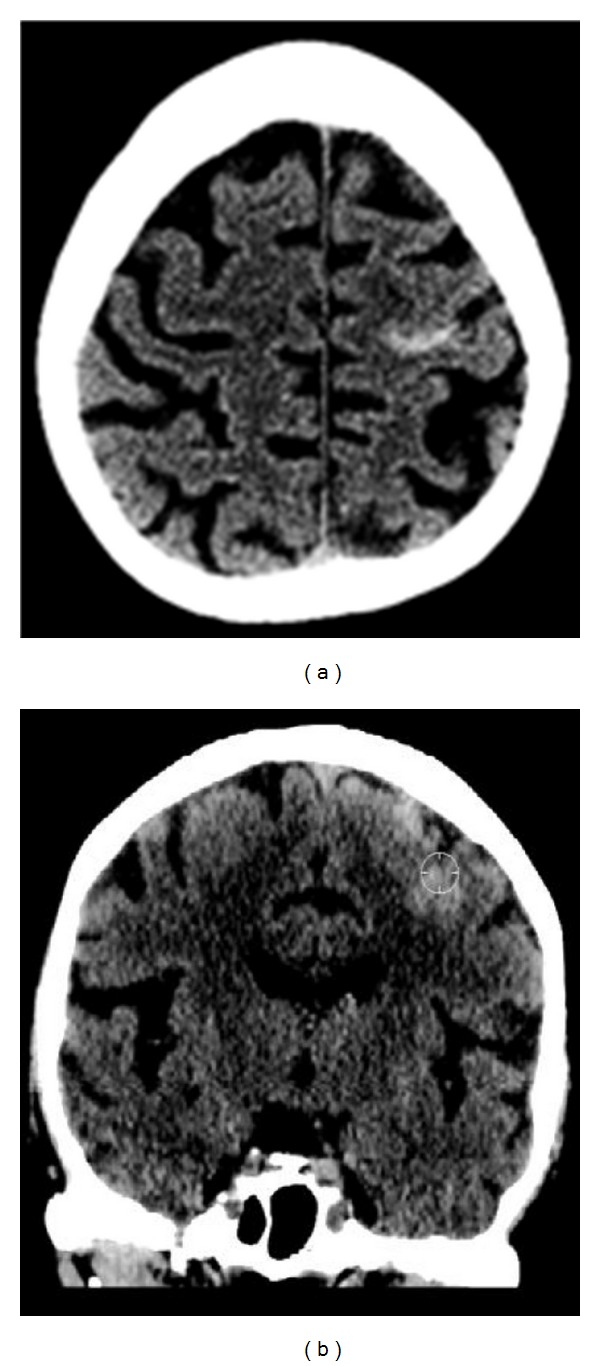
Unenhanced CT images of the brain. Axial and coronal CT images ((a) and (b)) of the brain in an 84-year-old normotensive female without history of trauma demonstrate a linear area of increased attenuation within the left frontal convexity, consistent with an isolated central sulcus hemorrhage.

**Figure 2:**
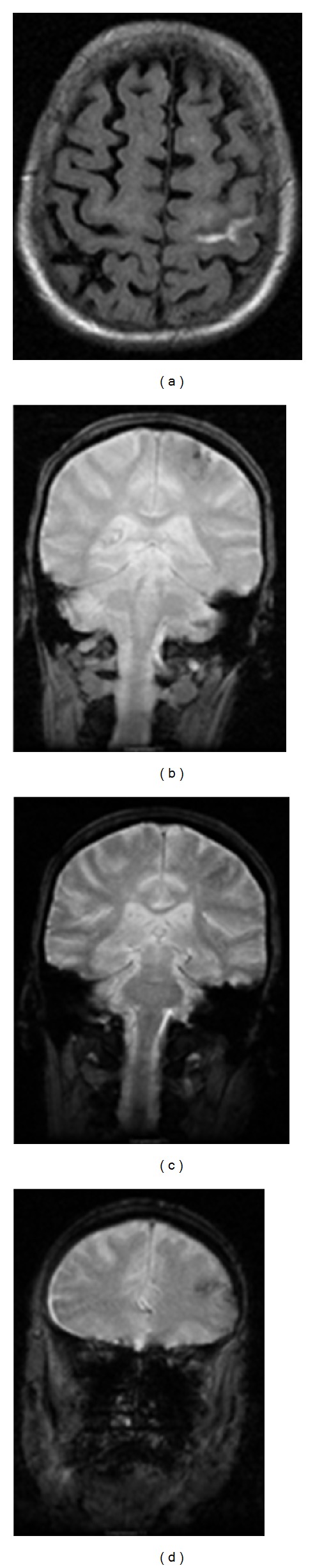
MR images of the brain. Axial FLAIR image (image a) of the index case confirms the finding of left central sulcus hemorrhage seen on the CT scan. Coronal gradient echo images (image b) demonstrate corresponding loss of signal within the left central sulcus. Coronal gradient echo images (images c, d) demonstrate signal loss within other adjacent cortical and subcortical areas consistent with hemorrhages and siderosis. Imaging findings and clinical presentation support the working diagnosis of cerebral amyloid angiopathy.

**Figure 3:**
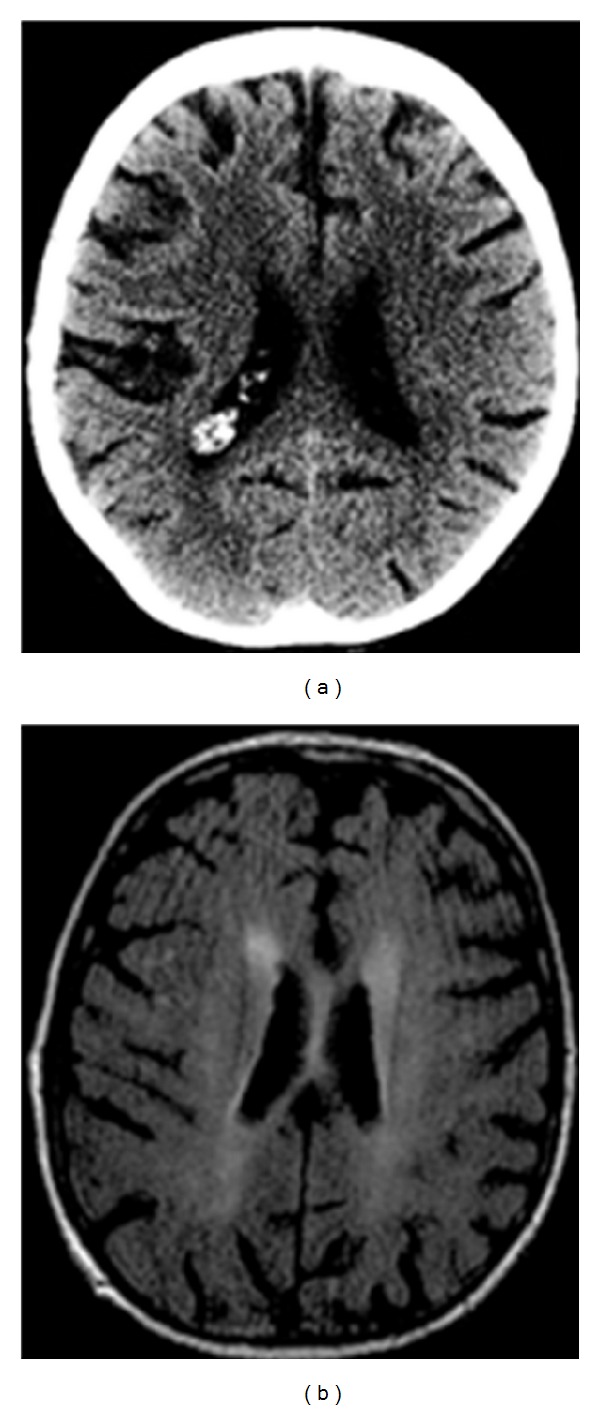
Associated findings. Unenhanced CT and MR FLAIR images of the brain demonstrate non-specific white matter related changes that can be associated with peripheral hemorrhages in cerebral amyloidosis.

Unenhanced computed tomographic (CT) head scan demonstrated increased attenuation along the left cerebral convexity consistent with isolated central sulcus hemorrhage without any mass effect or midline shift (Figure 1). Magnetic resonance (MR) imaging confirmed isolated central sulcus subarachnoid hemorrhage and also demonstrated adjacent areas of superficial siderosis and cortical/subcortical hemorrhages within the left cerebral hemisphere (Figure 2). Nonspecific white matter changes were also noted (Figure 3). Subsequent CT and MR angiograms were unremarkable without any aneurysms or vascular malformations. A working diagnosis of CAA was made based on Boston criteria [1]. Patient was monitored and discharged after stable hospital course with further clinic followup and instructions to avoid anticoagulation and antiplatelet agents. Follow-up CT head scan demonstrated interval resolution of the left central sulcus hemorrhage.

**Figure 4:**
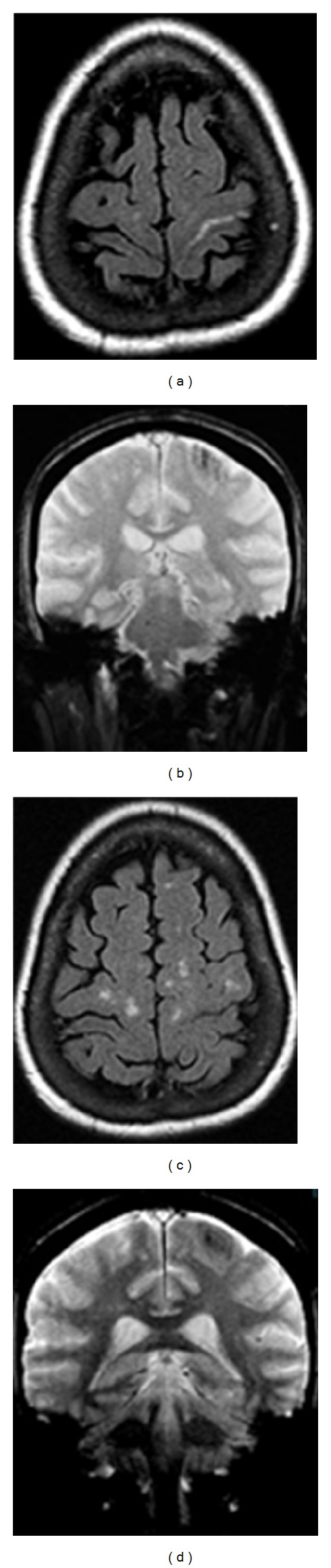
Second case of isolated central sulcus hemorrhage. MR images in a 67-year-old female with history of nasal cavity lymphoma presenting with right sided weakness. There is no history of trauma, hypertension, or bleeding diathesis. Flair MRI (image (a)) demonstrates isolated increased signal within the left central sulcus with corresponding loss of signal on gradient echo (image (b)) consistent with isolated central sulcus hemorrhage. Nonspecific white matter changes are seen on the FLAIR sequence (image (c)). MR angiogram did not demonstrate any aneurysms or vascular malformations. A probable diagnosis of cerebral amyloidosis was made based on the Boston criteria. Follow up MR images demonstrated interval resolution of the acute hemorrhage with chronic cortical and subcortical changes on the gradient echo sequences (image (d)).

**Figure 5:**
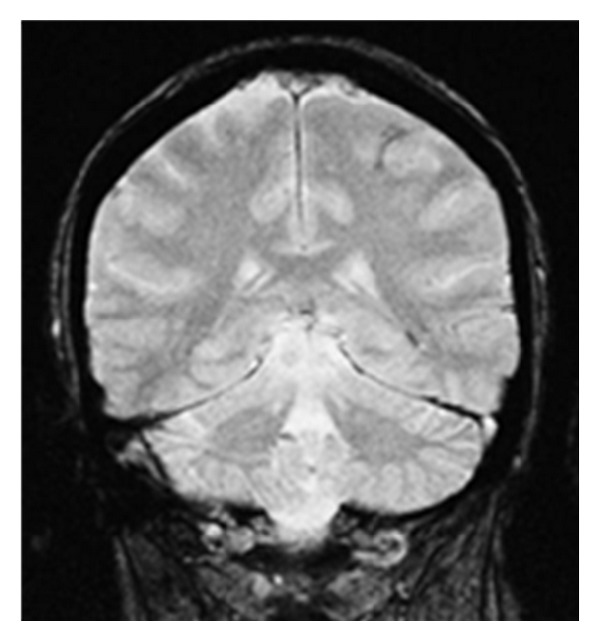
Third case of isolated central sulcus hemorrhage. Gradient echo MR images in a 50-year-old female with history of renal transplant and presenting with left leg weakness demonstrated signal loss within the left central sulcus consistent with an isolated hemorrhage. Associated nonspecific white matter changes were also seen. Angiographic work up was without any evidence of aneurysms or vascular malformations. Although the age of the patient and absence of other areas of chronic hemorrhage on MR imaging make this an atypical presentation, findings of an isolated central sulcus hemorrhage in a normotensive patient without other etiologies accounting for that hemorrhage would suggest a possible diagnosis of CAA.

Other similar cases with findings of isolated central sulcus hemorrhage and a diagnosis of cerebral amyloidosis based on Boston criteria are illustrated in Figures 4 and 5.

## 3. Discussion

Various etiologies summarized in Table 1 can present with intracranial hemorrhage, especially within the cortical or subcortical areas on imaging [2–5]. The pattern of bleeding in CAA is peripheral, cortical or subcortical, micro-and macrohemorrhages with sparing of the deep white matter, basal ganglia, and thalami [6]. Cerebellar and intraventricular involvement is rare. The common etiologies for hemorrhage in a non-traumatic setting are usually related to hypertension and aneurysms. Hypertensive hemorrhages occur centrally within the thalamus and basal ganglia. Cerebral aneurysms are more common with a family history and present with rupture and bleeding, usually within the vicinity of the aneurysm. Trauma related hemorrhages are usually seen within the inferior frontal and temporal regions that are prone to contusion and are associated with epidural or subdural hematomas in the region of coup and contrecoup injuries. Vascular malformations present as isolated cortical or extra-axial hemorrhages. CT or MR angiograms help in characterization of possible causes of peripheral intracranial hemorrhages such as aneurysms and vascular malformations.

Cerebral amyloidosis is one of the etiologies for spontaneous non-traumatic cortical or subcortical bleed in a normotensive patient. Amyloid deposition in the brain occurs within various pathologies such as Alzheimer's dementia, Creutzfeld Jacobs's disease, spongiform encephalopathies, and postradiation necrosis or can be rarely hereditary [6]. Deposition of amyloid within the cortical, subcortical, and leptomeningeal cerebral vessels results in increased fragility, hemorrhages, microaneurysms, and vascular irregularity or stenosis [7, 8]. Cerebral amyloidosis can be asymptomatic or clinically present with symptoms related to acute or chronic hemorrhage and ischemia. 

Cerebral amyloidosis commonly presents as peripheral cortical or subcortical hemorrhage but other rare and nonspecific patterns have been described in Table 2 [9–12]. The finding of a central sulcus hemorrhage is rare, but reported to be most frequently associated with amyloid angiopathy [13, 14]. The imaging modalities utilized during evaluation of an extra-axial or cortical-subcortical hemorrhage are described in Table 3. An acute hemorrhage can be identified easily on noncontrast CT imaging and is often the first imaging modality of choice. MR imaging, especially FLAIR (fluid attenuation inversion recovery) and gradient echo (GRE) or susceptibility weighted imaging (SWI) are helpful for confirmation and characterization of smaller cortical or subcortical bleeds. It also helps to identify other areas of chronic involvement. CT and MR angiograms exclude other common etiologies with cortical or subcortical bleeding. Boston criteria provide integration and standardization of clinical and imaging findings with diagnostic categorization (definite, probable with pathological evidence, and probable and possible diagnoses) based on clinical, histopathological, and MRI findings [1]. A biopsy is not usually warranted, and if obtained, staining of the amyloid with Congo red under polarized light demonstrates characteristic yellow green birefringence. Radiological work up in an elderly non-traumatic normotensive patient with cortical or subcortical hemorrhage excludes other causes of hemorrhage. Imaging in conjunction with clinical presentation establishes a putative diagnosis of cerebral amyloidosis. Management is currently limited and relies on optimal control of blood pressure, use of corticosteroids, and avoidance of antiplatelet agents and Warfarin [5, 15, 16]. Further understanding of the molecular pathogenesis would elucidate the role of immunosuppressants and lipid lowering drugs and help in the development of disease modifying therapies [8].

## 4. Conclusion

 Imaging plays an important role in recognition of typical patterns of various types of intracranial hemorrhage. It complements the clinical diagnosis and guides further management. Cerebral amyloidosis is a diagnosis of exclusion with varied clinical and imaging presentations. The typical imaging findings include a peripheral cortical or subcortical hemorrhage with other areas of chronic hemorrhage in an elderly, normotensive, and non-traumatic setting. As presented in our cases, an isolated central sulcus hemorrhage is rare and is reported to be most frequently associated with cerebral amyloidosis. Although histopathological diagnosis is seldom pursued, an isolated central sulcus hemorrhage may suggest a putative diagnosis of cerebral amyloid angiopathy, especially when further imaging and clinical presentation exclude alternative diagnoses.

## Figures and Tables

**Table 1:** Etiologies that can present with cortical, subcortical, or sulcal hemorrhage [2–5].

(i) Amyloid angiopathy (cortical/subcortical in location, may be associated with subarachnoid and subdural hemorrhages)	
(ii) Aneurysm rupture (subarachnoid and cisternal)	
(iii) Arterial dissection (subarachnoid hemorrhage, majority involve the posterior circulation)	
(iv) Bleeding diathesis (may show fluid-blood levels, associated with thrombocytopenia or abnormal prothrombin time)	
(v) Drug abuse (intraparenchymal or subarachnoid hemorrhage)	
(vi) Hypertension (central, involving the thalamus and basal ganglia)	
(vii) Malignancy (subcortical, associated edema and mass effect)	
(viii) Posterior reversible encephalopathy syndrome or PRES (focal intracerebral and subarachnoid hemorrhage with characteristic signal changes)	
(ix) Trauma (predilection for inferior frontal and temporal lobes)	
(x) Vascular malformations (subarachnoid or cortical hemorrhages, better characterized on CT or MR angiograms)	
(xi) Vasculitis (intraparenchymal and associated with multiple areas of subcortical infarctions)	
(xii) Venous thrombosis (subcortical)	

**Table 2:** Imaging presentations of cerebral amyloidosis [9–12].

(i) Intracranial hemorrhage:	
(a) Acute and chronic cortical, subcortical, and rarely intraventricular	
(b) Spares the deep white matter, thalamus, and basal ganglia	
(c) Central sulcus hemorrhage	
(d) Characteristically multiple, bilateral, peripheral, and lobulated hemorrhages with coexisting old hemorrhages support the diagnosis	
(ii) Leukoencephalopathy	
(iii) Atrophy and cerebral volume loss	
(iv) Vascular luminal narrowing and ischemia	
(v) Amyloidoma simulating a mass	

**Table 3:** Imaging modalities for evaluation of peripheral intracranial hemorrhage.

(i) Non-contrast CT: initial test of choice	
(ii) MRI:	
(a) FLAIR—acute or subacute hemorrhage (non-specific)	
(b) GRE or SWI—decreased signal and blooming in areas of prior hemorrhage	
(iii) CT and MR angiograms: diagnosis and characterization of aneurysms, AVM, and vasculitits	
(iv) Angiography: limited value, invasive procedure	

## Abbreviations

AVM: Arteriovenous malformation
CAA: Cerebral amyloid angiopathy
CT: Computed tomography
FLAIR: Fluid attenuation inversion recovery
GRE: Gradient echo
MRI: Magnetic resonance imaging
PRES: Posterior reversible encephalopathy syndrome
SWI: Susceptibility weighted imaging.
